# Oxidative Stress and Free Radical Processes in Tumor and Non-Tumor Obstructive Jaundice: Influence of Disease Duration, Severity and Surgical Treatment on Outcomes

**DOI:** 10.3390/pathophysiology29010005

**Published:** 2022-01-31

**Authors:** Ekaterina Vladimirovna Silina, Victor Alexandrovich Stupin, Igor Sergeevich Abramov, Sergey Brankovich Bolevich, Gouri Deshpande, Raghu Ram Achar, Tatiana Georgievna Sinelnikova

**Affiliations:** 1 Department of Human Pathology, I.M. Sechenov First Moscow State Medical University (Sechenov University), 119991 Moscow, Russia; bolevich2011@yandex.ru (S.B.B.); sintatiana@rambler.ru (T.G.S.); 2 Department of Hospital Surgery No. 1, N.I. Pirogov Russian National Research Medical University, 117997 Moscow, Russia; stvictor@bk.ru (V.A.S.); abramov-1961@mail.ru (I.S.A.); 3 Division of Biochemistry, School of Life Sciences, JSS Academy of Higher Education & Research, Mysuru 570 015, India; gourideshpande91@gmail.com (G.D.); rracharya@jssuni.edu.in (R.R.A.); 4 Department of Biochemisty, Karnatak University, Dharwad 580 003, India

**Keywords:** free radical processes, obstructive jaundice, tumor jaundice, non-tumor jaundice, oxidative stress, reactive oxygen species, malondialdehyde, markers of severity, prognosis

## Abstract

The aim of this study was to assess the patterns and pattern disruptions of free radical processes in patients with obstructive jaundice of various origins, and the severity of jaundice before and after decompression. Oxidative stress markers were determined in 128 patients with obstructive jaundice with a tumor genesis (23.4%) or non-tumor genesis (76.6%). The patients were hospitalized at different stages of clinical signs of jaundice. We studied the anti-peroxide activity in plasma, basal and stimulated indicators of the chemiluminescence intensity in leukocytes, leukocyte activity coefficients reflecting the level of reactive oxygen species generated by leukocytes, malondialdehyde levels indicative of the degree of lipid peroxidation and cellular destruction, liver enzymes (markers of cytolysis) and bilirubin levels. Data for hepatocyte death and markers of oxidative stress correlated with the severity of jaundice, its duration and the method of its surgical correction. It is proposed that using markers of free radical processes to assess the prognosis and effectiveness of treatment and to personalize treatment measures will improve the results of jaundice treatment.

## 1. Introduction

Obstructive jaundice is one of the five most common syndromes encountered in surgical practice. Jaundice develops primarily as the result of cholelithiasis with choledocholithiasis, stenoses of the distal common bile duct and tumors of the pancreatic head. Cholelithiasis is the most frequent cause [1,2,3]. A predictable number (10–15%) of patients with stones in the gallbladder have choledocholithiasis. Patients with isolated choledocholithiasis, i.e., without stones in the bladder, are most often found in Southeast Asia. The stones differ in terms of chemical composition, meaning that the stones may occur for various reasons. Gallstone disease affects 10–20% of the US adult population, where more than 720,000 cholecystectomies are performed every year [4]. However, since no studies exist that analyze the reasons why stones appear and develop, there is no discussion of treatment strategies. Several papers have documented individualization of treatment approaches in patients with stones. Gallstone disease occurs in 60–70% of Native American patients in North America, but decreases significantly in the offspring of mixed marriages [5]. Physical activity reduces the risk of gallstone disease [6], while metabolic disorders of various kinds increase the risk [7]. There are reliable observations of the effect of colectomy surgery on the appearance of calculi in the course of ulcerative colitis [8]. The presence of calculi in a gallbladder and duct system can only be considered a disease if it has clear etiopathogenetic roots and should rightfully be recognized as a syndrome. Perhaps the only thing that all researchers agree on is that women are more likely to develop gallstones. By the age of 60, almost 25% of women have gallstones; in women aged 75 years and older, stones are detected in every second woman [3,9]. Considering the increasing incidence of complicated gallstone disease, the number of reviews devoted to this problem is growing in parallel with the problem itself.

The development of modern instrumental diagnostic methods, including ultrasound, endoscopic retrograde cholangiography, nuclear magnetic resonance and multispiral computed tomography, allows doctors to identify the cause of jaundice and choose the optimal surgical intervention on the first day of treatment. However, advances in diagnostics and surgery over the past decade have not dramatically improved treatment results, and the mortality rates for the syndrome persist, ranging from 1% for uncomplicated to 45% for complicated cases, complications during treatment or severe comorbidity [10,11,12]. Postoperative mortality among patients with non-neoplastic jaundice varies from 4% to 23% [13,14]; in patients with neoplastic jaundice, it ranges from 13% to 60% [15,16,17]. The persistent mortality rates may be the reason why increased attention is being paid to the study of pathophysiological processes, not only those that occur specifically in the liver but also general processes affecting other organs and systems. These studies aim to determine the optimal management of patients in the perioperative period and the practical implementation of personalized medicine.

During the first 3–5 days of obstructive jaundice, the permeability of hepatocyte membranes increases, indicator enzymes are released and the hepatocytes continue to excrete bile. As the increased pressure in the bile ducts continues, it leads to dysfunction of liver cells, increased levels of unconjugated bilirubin in the plasma, onset of necrosis in the hepatocytes and increased activity of aminotransferases [18,19]. However, the question that needs to be raised is why acutely emerging jaundice quickly causes acute hepatic and renal failure, while patients with slowly developing jaundice, typical of jaundice caused by tumors, can live for many months without suffering critical damage.

The changes caused by jaundice disrupt the antitoxic function of the liver, which is accompanied by endotoxemia. Microvascular thrombosis develops in the kidneys, the levels of urea and creatinine increase in the blood and disruptions in the liver increase. Toxic substances penetrate the blood-brain barrier, causing hepatic encephalopathy to develop [20]. Cholestasis has a damaging effect on both the tubular epithelium and hepatocytes [21]. The bile components (hydrophobic bile acids, bilirubin, cholesterol) present in high concentrations in obstructive jaundice have a toxic effect on hepatocyte mitochondria, directly or indirectly blocking the respiratory cycle and fatty acid oxidation [22]. The result is further dysfunction of the hepatocytes and stimulation of lipid peroxidation processes, which leads to rapid cell death and liver cirrhosis [23].

We assume that free radical processes commonly occur during the course of polyetiologic obstructive jaundice and miscoordination of cellular metabolic processes. For this reason, markers of reactive oxygen species and lipid peroxidation can be used as objective indicators of the severity of disease and for prediction of its successful or unsuccessful outcome. We decided to test this hypothesis for obstructive jaundice in patients of different ages with various etiologies, severities and durations of obstructive jaundice.

The aim of the study was to assess the patterns and pattern disruptions of free radical processes in patients with obstructive jaundice of various tumorous and non-tumorous genesis, and the severity of the jaundice before and after decompression.

## 2. Materials and Methods

This study was approved by the Independent Ethics Committee JSC Group of Medical Clinics ‘Medsi’ (protocol No. 83, dated 28 Dec 2020; Moscow, St. Krasnaya Presnya, 16).

This study did not provide an additional intervention and complied with all ethical standards. During the study, the personal data of the patients were not disclosed. The inclusion of a patient in the study did not lead to any changes in the general methods of treatment or the regimens and/or doses of the drugs used, which corresponded to the standards of medical care.

The study was carried out in 128 patients (54 men (42.2%) and 74 women (57.8%)) with various pathologies of the hepato-pancreatic-duodenal zone (HPDZ) complicated by obstructive jaundice, who were hospitalized in surgical and intensive care units. The age of the patients ranged from 21 to 90 years (Me = 72 years, interquartile range [57:80] years).

Obstructive jaundice developed in these patients for the following reasons: diseases with a tumor genesis (30 patients; 23.4%) or with a non-tumor genesis (98 patients; 76.6%). The oncological genesis of the obstructive jaundice was established in tumors of the pancreas and/or Vater’s papilla (*n* = 22), and less often, tumors of the biliary system (*n* = 8), including a tumor of the gallbladder (*n* = 3) and metastases in the liver gate from malignant tumors of other organs (*n* = 5).

Pathologically, tumor jaundices were caused by adenocarcinomas. Tumors of the pancreas were represented by intraductal adenocarcinomas, the histological form of cancer of the major duodenal papilla was also adenocarcinoma and the tumors of the gallbladder were adenocarcinoma in all cases. Metastases in the liver gate, meanwhile, were gastric adenocarcinoma and pancreatic adenocarcinoma. We did not study the molecular basis of tumors since the treatment was carried out not in a specialized oncological, but in a general surgical hospital, and operations were performed on patients for vital urgent indications.

**Figure 1:**
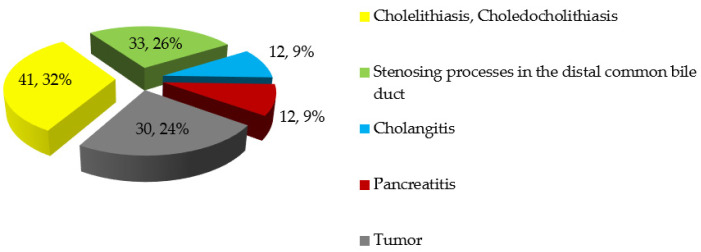
Distribution of patients by the pathologies responsible for the development of obstructive jaundice (abs, %).

Non-tumorous jaundice was subdivided according to the leading causes as follows. The most common cause of non-tumorous jaundice was cholelithiasis with choledocholithiasis (*n* = 41; 41.8%). In addition, there were various chronic processes in the distal bile duct (*n* = 33; 33.7%), including stenosis of the common bile duct, diverticulum adjacent to the major duodenal papilla and other chronic pathologies of the major duodenal papilla. Cholangitis was the main complication of jaundice in 12 patients (12; 2%) while acute pancreatitis was the cause in 12 patients with non-neoplastic jaundice (12; 2%). The frequency distribution of the main causes of obstructive jaundice in patients included in the study is shown in Figure 1. It is important to note that in 49 cases (38.3%), jaundice could have been caused by several factors simultaneously. For example, in conjunction with cholelithiasis, choledocholithiasis and diverticulum adjacent to the major duodenal papilla were detected in 12 patients with non-tumorous jaundice (12.2%). Previously, 16 patients (12.5%) (12 with non-tumorous and 4 with tumorous jaundice) underwent cholecystectomy.

On hospitalization, the levels of biochemical markers of cholestasis (bilirubin, alkaline phosphatase, gamma-glutamyl transferase) were significantly high. The total level of bilirubin during hospitalization exceeded the upper limit of normal in all patients and varied considerably from 22 to 647 μmol/L (Me = 118 μmol/L, interquartile range 74:186 μmol/L). Total bilirubin levels of less than 100 μmol/L during hospitalization were recorded in 50 patients (39.1%), while levels that ranged from 101 to 150 μmol/L were recorded in 36 patients (28.1%). In 42 cases (32.8%), the total bilirubin level was higher than 150 mmol/L. The latter values were from patients with tumorous jaundice, which is characterized by bilirubin levels on average 1.6-fold higher than those in non-tumorous jaundice (177 [108:297] μM/L versus 110 [71:148] μM/L; *p* < 0.01). The levels of alkaline phosphatase (AP) recorded during hospitalization were five-fold higher on average than the upper limit of normal, reaching 673 [452:959] U/L. In tumorous jaundice, the AP levels were on average 1.45-fold higher than those for non-tumorous jaundice (914 [569:1269] U/L versus 629 [388:880] U/L; *p* = 0.001).

The levels of gamma-glutamyltransferase (GGT) were 10-fold higher than the upper limit of normal, averaging 419 [203:634] U/L. Comparative analysis of this indicator during hospitalization of patients with tumorous and non-tumorous jaundice did not reveal statistically significant differences (*p* > 0.05).

The levels of the cytolysis markers alanine aminotransferase (ALT) and aspartate aminotransferase (AST) also significantly exceeded the normal limits. During hospitalization, the average ALT (Me) was 191 U/L (interquartile range 114:290), which is six-fold greater than the upper limit of normal, while the AST indicator exceeded the upper limit of normal by three-fold on average (Me = 119 U/L; interquartile range 82:201).

The severity of a patient’s disease was assessed using SOFA (the Sepsis-Related Organ Failure Assessment score), which ranged from two to eight points. There were 77 patients (60.2%) with scores of moderate severity (less than four points on the SOFA scale) and 51 patients (39.8%) with scores of high severity (four or more points on the SOFA scale). There were 2.5-fold more patients with tumorous jaundice who had severe scores (≥4 on the SOFA scale) than patients with non-tumorous jaundice (73.3% versus 29.6%, *p* <0.01).

The duration of jaundice (calculated from when a patient entered hospitalization) ranged from 1 to 60 days; 34.4% of patients (*n* = 44) were hospitalized within 48 h after the onset of jaundice, 40.6% (*n* = 52) were admitted within 3–6 days after the onset of jaundice and 25% (*n* = 32) had the disease for seven or more days before hospitalization. Early hospitalization of patients with symptoms of jaundice was typical for non-tumorous jaundice (41.8%), timely hospitalization was provided to patients with acute pain syndrome and the most time elapsed for patients with tumorous jaundice (56.7%). These figures statistically distinguish the types of jaundice by their genesis (*p* < 0.01).

Surgical treatment was carried out after establishing the type of jaundice according to the clinically accepted procedures, which aimed at the earliest possible elimination of hypertension from the gallbladder with the implementation of minimally invasive decompression of the biliary tract. Surgical interventions were performed on 100 patients (78.1%), while 28 patients (21.9%) were not operated on for various reasons (refusal of the operation; unaided separation of calculus during the endoscopic examination; severity of the condition, e.g., inoperable tumors with damage to the liver parenchyma) but received conservative therapy. There was no statistically significant difference in the frequency of surgical or conservative therapy (*p* > 0.05) between the tumorous and non-tumorous jaundice groups. The most frequent operation (*n* = 57; 44.5%) was endoscopic papilosphincterotomy (EPST) with the removal of calculus, which was performed in 58.2% of patients with non-tumorous jaundice (this procedure is not performed for patients with tumors). Microcholecystostomy (puncture of the gallbladder) was performed on 15 patients (11.7%), most often in those with tumors of the pancreatic head (*n* = 9; 30% of operations performed in patients with tumorous obstructive jaundice). Cholecystectomy with drainage of the bile duct was performed for 17 patients (13.3%), most often those with tumors or with destructive cholecystitis (9.2% in patients with non-tumorous and 26.7% in patients with tumorous jaundice).

Nasobiliary drainage and gall bladder stenting were performed least often, in only 8.6% of patients (more often for patients with tumors: 4.1% of patients with non-tumorous jaundice compared with 23.3% of patients with tumorous jaundice).

Decompressional operations were performed after establishing the cause of the jaundice and preparing the patient for surgery. On average (median), operations were performed on the second day of hospitalization (interquartile range [1:3] day); 78% of operations were performed within 72 h from the time of admission to the hospital.

During hospitalization, all patients received standard intensive drug therapy, including fluid infusions, antibiotics, antispasmodics and pain relievers. Patients with jaundice who received extracorporeal detoxification treatment in addition to the standard of care were not included in this analysis.

The distribution of patients by sex, age, duration and severity of jaundice (tumor or non-tumor genesis) is presented in Table 1.

Patient condition assessment was carried periodically (at the time of admission and on the 3rd, 8th and 14th days after the removal of the biliary tree block). This included complete and biochemical blood counts, an assessment of disease severity according to the SOFA scale [24], ultrasound examination of the hepatic-pancreatic-duodenal zone organs—using Philips Epiq Elite (Koninklijke Philips NV, Amsterdam, The Netherlands), GE LOGIQ E9 (General Electric Healthcare, Chicago, IL, USA) Esaote Mylab devices (Esaote, Genoa, Italy)—and endoscopic retrograde cholangiopancreatography (ERCP) performed using Q190V (Olympus Medical, Tokyo, Japan) and Pentax ED-3430TK (Pentax Medical, Tokyo, Japan) video duodenoscopes. Some patients underwent computed tomography if indicated.

Laboratory parameters were determined after taking blood from the ulnar vein on the first day of hospitalization. In addition to analyzing the standard blood biochemical parameters (total bilirubin, alkaline phosphatase, GGT, ALT, AST, etc.), we assessed the indices of free radical processes (FRP) using methods described previously [25,26,27,28]. FRP include malondialdehyde (MDA) and luminescence indices: basal chemiluminescence intensity indicator (bCLII), zymosan-stimulated chemiluminescence intensity indicator (sCLII), leukocyte activity coefficient (AC) and anti-peroxide plasma activity (APA).

We performed luminescence assessment of the cell cultures and plasma using an LKB WALLAC Luminometer (LKB Instruments, Mount Waverley, Victoria, Australia). After centrifuging the blood to isolate the leukocyte sediment, the solution was resuspended in 1 mL of an isotonic solution of 0.9% NaCl and a phosphate-alkaline buffer solution (7.5 mL + 2.5 mL, respectively) with a pH of 7.35. This produced a leukocyte mass consisting of only phagocytes (granulocytes and monocytes) and lymphocytes. After isolating the leukocyte mass (0.2 mL with a standard concentration of leukocytes: 2500 leukocytes in 1 μL), the chemiluminescence intensity indicators (CLII) demonstrating the generation of reactive oxygen species by blood leukocytes were determined according to the formula:

(1) $$\mathrm{CLII}=\frac{\mathrm{maximal}\;\mathrm{chemiluminiscence}\times 1,000,000}{\mathrm{numberof}\;\mathrm{granulocytes}\;\mathrm{and}\;\mathrm{monocytes}\;\mathrm{in}\;a\;\mathrm{leukocyte}\;\mathrm{mass}}$$

The results are expressed in mV/s × 10^6^ leukocytes (mV/s × 10^6^ leukocytes).

Chemiluminescence was recorded before reaching the first maximum, thus demonstrating the basal chemiluminescence intensity index (bCLII), an integral indicator characterizing the spontaneous chemiluminescence of leukocytes at rest without stimulation. At the maximum chemiluminescence, phagocytosed corpuscular particles of the activator were added (1% solution of opsonized zymosan; 1 leukocyte to 100 parts of zymosan tightly bound to complement), and the maximum luminescence level was recorded within 10 s. These numbers represent the stimulated chemiluminescence intensity index (sCLII), which reflects the level of generation of reactive oxygen species by leukocytes (phagocytes) during their stimulation and restructuring of oxidative metabolism. This action is necessary for leukocytes to perform their phagocytic function during bacterial invasion or when large molecular decomposition products are encountered in the tissues or bloodstream.

After measuring bCLII and sCLII, the leukocyte activity coefficient (AC) was calculated using the formula: sCLII/bCLII. The result corresponds to the rate at which stimulated generation of reactive oxygen species and phagocytosis exceeds the rate of the basal equilibrium metabolic activity of leukocytes, which reflects free radical metabolic rearrangement of leukocytes.

The indicator of anti-peroxide plasma activity (APA) reflects the level of response of its antioxidant systems and is a calculated indicator. The method for determining the APA index is based on measuring and determining the ratio of spontaneous and peroxide-induced chemiluminescence of secondary plasma. The secondary plasma (without cells) was obtained by double centrifugation at 3000 rpm. The parameters of hydrogen peroxide-induced plasma chemiluminescence and spontaneous plasma chemiluminescence at rest were then compared. The spontaneous chemiluminescence of the secondary plasma reflected the luminescent response of the plasma at rest. The induced chemiluminescence of the secondary plasma reflected the plasma response after the addition of 0.05 mL 3% H_2_O_2_ solution. The APA indicator is expressed in relative units (RU).

Malondialdehyde (malonic dialdehyde; MDA) is a secondary product of lipid peroxidation, characterizing the level of cell intoxication and death [25,26,27,29,30]. The MDA titer was determined in secondary plasma via its reaction with 2-thiobarbituric acid and the formation of a chromogen complex with an absorption maximum in the red region of the visible spectrum (532 nm). The optical density was measured on a spectrophotometer at a wavelength of 532 nm. The concentration of the MDA plasma was calculated by the formula below, taking into account the molar extinction coefficient of the formed complex, which is equal to 1.56 × 10^5^ M^−1^ cm^−1^. The final result was expressed in micromoles per liter of plasma (μM/L).

(2) $$C=\frac{D\times V^{p}\times 10^{6}}{\varepsilon }$$

where *C* is the concentration of the MDA plasma, *D* is the optical density at a wavelength of 532 nm, *V* is the volume of the reaction mixture, *p* is the dilution factor, 10^6^ is the conversion factor in μM and ε is the coefficient of molar extinction of the MDA complex with 2-thiobarbituric acid (1.56 × 10^5^ M^−1^ cm^−1^).

The FRP were studied in all (100%) patients at the time of admission to the hospital. In dynamics, a smaller number of patients were examined due to discharge from the hospital, transfer to other departments, unfavorable outcomes and other reasons. On the third day after decompression, the MDA plasma values were determined in 99 patients, and the other FRP values were recorded in 88 patients. On the eighth day after decompression, the MDA plasma value was determined in 86 patients and the other FRP values were recorded in 74 patients. On the 14th day, the MDA plasma value was determined in 50 patients and the other FRP values were recorded in 40 patients. The values of FRP parameters in patients with jaundice were compared with those of healthy volunteers (*n* = 33).

Statistical analysis of the research results was carried out using SPSS 23.0. Descriptive statistics of continuous quantitative indicators are presented as the mean (M), standard deviation (±SD), standard error of the mean (m) with a normal distribution, median (Me) and the values of the lower (25%) and upper (75%) quartiles with a distribution other than normal. The interquartile range in the tables is enclosed in square brackets including Tukey’s HSD test. The distribution normality was assessed using the Kolmogorov-Smirnov test. Correlation analysis was carried out according to Pearson’s and Spearman’s methods and included assessment of the two-sided criterion of statistical significance. To compare two independent nonparametric samples, the Mann-Whitney U test was used. For multiple comparisons, the Kruskal-Wallis test was used. The Wilcoxon test was used to compare two dependent nonparametric samples, and the Friedman test was used for multiple comparisons. Qualitative variables were compared using the χ^2^ test (analysis of contingency tables). Differences were considered statistically significant at *p* < 0.05.

## 3. Results

### 3.1. Study of Oxidative Stress Indicators on the First Day of Hospitalization of Patients with Obstructive Jaundice of Varying Severity, Duration and Genesis

We found that all indicators of FRP for patients with obstructive jaundice differed in a statistically significant manner from those of healthy individuals on the first day of hospitalization. Then, depending on the duration of jaundice and its severity, these differences continued to vary and increase. For patients with jaundice, the AC indicator was significantly higher than normal (3.9-fold on average; *p* < 0.01) due to a significant increase in the sCLII indicator (3.2-fold on average; *p* < 0.01) and a decrease in the bCLII indicator (1.6-fold on average; *p* < 0.05), illustrating the limiting mobilization of phagocytic systems. The MDA index, which increased 3.8-fold compared to normal (*p* < 0.01), confirms this assumption. The level of plasma protective APA in patients with obstructive jaundice was 1.6-fold lower on average than normal (*p* < 0.01), which indicates insufficiency or depletion of their anti-peroxide systems. All the factors above demonstrate the important role of oxidative stress in the pathological physiology of jaundice and the feasibility of antioxidant pharmacotherapy while treating jaundice (Table 2).

Comparative analysis of FRP indices in patients with jaundice of tumorous and non-tumorous genesis revealed a distinct FRP imbalance in tumors. All the studied parameters of FRP deviated from the norm in tumor jaundice (on average, bCLII decreased 2.2-fold and APA 1.6-fold; sCLII indicators increased three-fold, AC four-fold and the MDA level increased 8.6-fold to 24.1 μmol/L (*p* < 0.01)). In patients with non-tumorous jaundice, the bCLII index did not deviate from normal. Changes in the other parameters were statistically significant (the APA index decreased 1.7-fold; sCLII increased 3.4-fold, AC 3.9-fold and the MDA level increased 3.2-fold). A statistically significant difference was recorded in the MDA level between the tumorous and non-tumorous jaundice groups; the level was 2.7-fold higher in the tumorous group on average (*p* < 0.01) (Table 3). This allows us to consider the MDA indicator for blood plasma as a possible clarifying criterion in the differential diagnosis of the jaundice etiology in a number of cases. This was confirmed by performing correlation analysis of the MDA levels and tumorous genesis of the jaundice (r = 0.507; *p* < 0.01).

In patients hospitalized with severe disease (more than four points on the SOFA scale), the level of total bilirubin in the blood plasma was significantly higher than in patients with moderately severe disease (*p* < 0.001) (Table 4).

The imbalance in summarily calculated indices demonstrating the greatest increase in sCLII and MDA and a decrease in APA correlated with the severity of the condition and was maximally expressed in severe patients who had ≥4 points on the SOFA scale upon hospitalization. The MDA level was on average 2.2-fold higher for severe patients compared with the moderately severe patients (Table 5). Consequently, the indicators characterizing the FRP can be used in clinical practice to assess the severity of the patient’s condition along with other scales.

The correlation analysis revealed a statistically significant positive correlation between the severity of the jaundice, assessed in points on the SOFA scale, and the MDA indicator (r = 0.418; *p* < 0.001). The severity of the disease also correlated directly with the level of total blood bilirubin (r = 0.536; *p* < 0.001).

**Figure 2:**
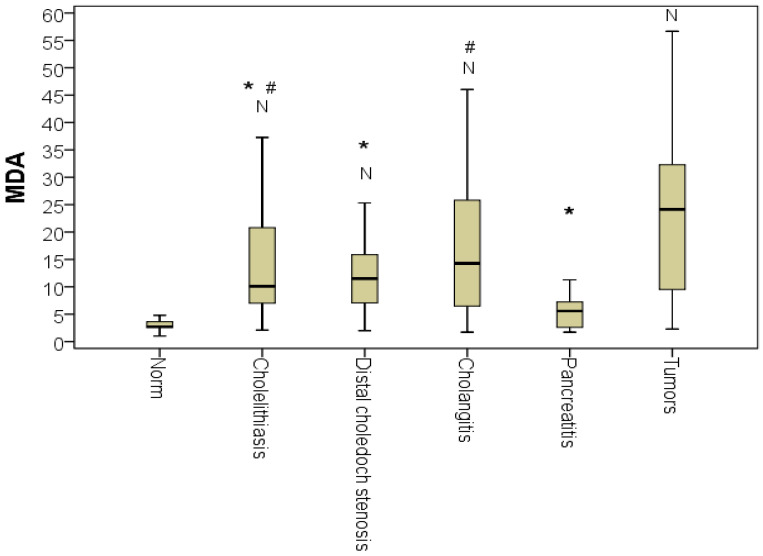
Malondialdehyde (MDA) indicator for different etiologies of jaundice (*n* = 128; *p* < 0.01). * the difference from tumorous jaundice; # the difference from patients with acute pancreatitis; N, the difference from the norm at *p* < 0.01 (Kruskal-Wallis test).

The comparative analysis of the MDA index for various jaundice etiologies revealed multiple differences according to the Kruskal-Wallis test (*p* < 0.01). The MDA level during tumorous jaundice was significantly higher than the MDA level during cholelithiasis and choledocholithiasis by 2.4-fold on average (*p* < 0.01). It was also higher than in chronic pathologies of the choledochal bile duct by 2.5-fold on average (*p* < 0.01). For the most part, tumorous jaundice MDA levels were higher compared to those of MDA of patients with jaundice as the result of acute pancreatitis (4.3-fold on average; *p* < 0.01). The MDA level in patients with cholangitis did not differ from the MDA level in patients with tumorous jaundice (*p* > 0.05). Thus, MDA correlated with the overall severity of the patient’s condition, regardless of the cause of the disease. We emphasize that the MDA of patients with acute pancreatitis did not statistically differ from normal on the first day of hospitalization, confirming the diagnostic importance of this marker (Figure 2).

**Figure 3:**
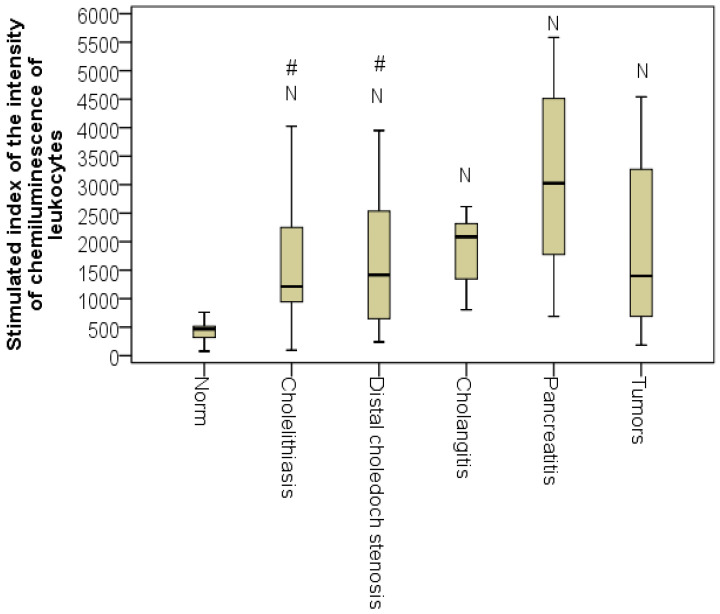
sCLII for different jaundice etiologies (*n* = 128; *p* < 0.01). N, the difference from the norm at *p* < 0.01; # the difference from patients with acute pancreatitis at *p* < 0.05 (Kruskal-Wallis test).

The figures for sCLII differed statistically from normal for all jaundice etiologies (*p* < 0.01, taking into account the control group). Thus, from the first day of hospitalization, the explosive activity of leukocytes was already apparent. The highest level of sCLII was recorded in patients with acute pancreatitis (Me = 3025 mV/s × 10^6^ L) and cholangitis, demonstrating the highest intensification of the generation of reactive oxygen species by leukocytes during hospitalization of these patients (Figure 3).

This is explained by the transition of macrophages into the active phase with the production of reactive oxygen species for effective implementation, which was confirmed by the increased values of sCLII in the same groups.

**Figure 4:**
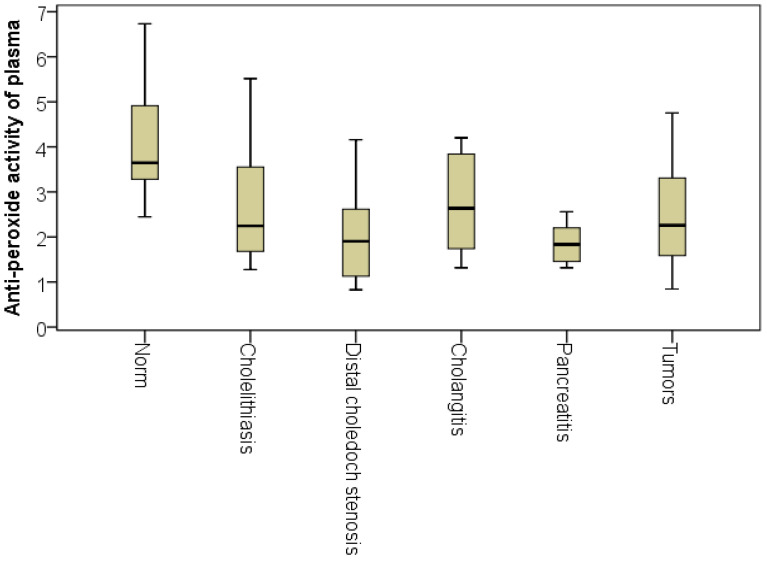
Plasma APA indices for various etiologies of obstructive jaundice.

However, at the same time, the patients with acute pancreatitis had the lowest APA level (Me = 1.8). This may be due to the rapidly developing enzymatic toxemia characteristic of this phase of acute pancreatitis, the complexity of the multicomponent anti-peroxide activity system’s development and/or the generalization of the problem. Despite the absence of significant intergroup differences, we noted that the highest APA level was recorded among the patients with cholangitis (Me = 2.6, which was on average 1.44-fold more than for patients with pancreatitis) (Figure 4). Analysis of the APA indicator percentiles with a step of 5% established that the normal APA plasma level (above 2.5 relative units) was observed in 60% of patients with cholangitis, 40% of patients with choledocholithiasis cholelithiasis, 40% of patients with tumor jaundice, 30% with chronic processes in the distal choledochal bile duct and in only 10% of patients with acute pancreatitis. It is very likely that the bacteremia that occurs during cholangitis is a ‘habitual,’ and therefore, easily recognizable trigger that begins the fight against microbial aggression. In addition, the duration of the anamnesis in these patients made it possible to assert with high reliability that by the time of hospitalization, both cellular and humoral immunities had been activated in patients. This conclusion is confirmed by the graph of APA indicators.

The study of FRP at different periods of cholestasis showed an increase in the imbalance over the course of jaundice. The most significant increase, which had already begun in the first hours of the disease, occurred in the MDA index (*p* < 0.001). Thus, the MDA level during the first 48 h of jaundice was 2.7-fold higher than normal (*p* < 0.01); on days 3–7, it was 3.5-fold higher (*p* < 0.01) and at the later stages of jaundice, it had increased by nine-fold on average (*p* < 0.01). The correlation analysis revealed a statistically significant positive correlation between the duration of the jaundice and the MDA index (r = 0.419; *p* < 0.001). Despite ongoing treatment, including decompression of the ductal system of the liver, such a long ‘tail’ of MDA indicates a rather slow process of inactivation and removal of destroyed hepatocytes from the bloodstream.

**Figure 5:**
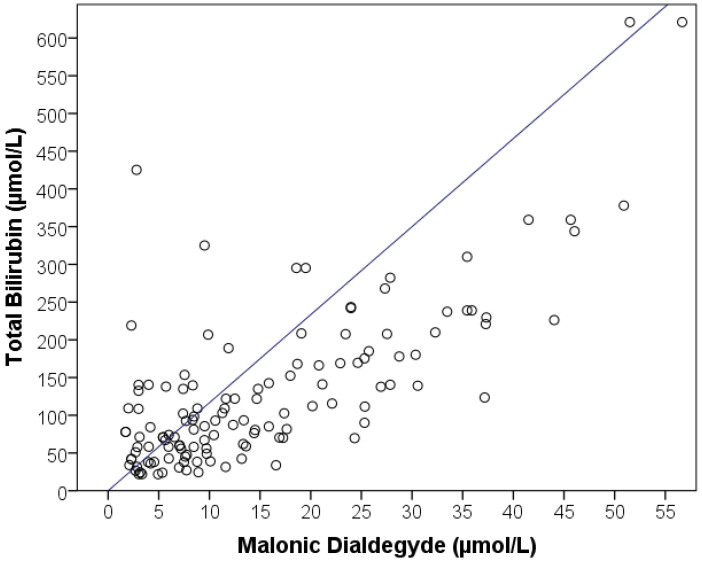
Correlation between the bilirubin level and concentration of MDA in blood plasma.

We analyzed the FRP indices in patients with different severities of hyperbilirubinemia, a standard laboratory indicator of the severity of jaundice. With an increase in the concentration of total bilirubin in the blood, a significant increase in the free radical imbalance was revealed, demonstrated by an increase in sCLII (*p* = 0.043), APA (*p* = 0.019), and MDA (*p* < 0.001) that either correlated with or outpaced blood bilirubin numbers. The most significant correlation was revealed in terms of MDA (r = 0.751; *p* <0.001) (Figure 5).

On average, the MDA level in patients with mild hyperbilirubinemia (less than 50 µmol/L) was 5.6 [3.2:8.8] µmol/L, which is two-fold higher than the norm. During hospitalization of patients whose total bilirubin level was 50–100 µmol/L, the MDA level in the blood plasma was measured at 8.4 [5.6:13.4] µmol/L on average, which is three-fold higher than normal and 1.5-fold higher than in patients with mild hyperbilirubinemia. The MDA level in patients with a high level of total bilirubin (100–200 µmol/L) averaged 15.9 [8.4:24.7] µmol/L, which is 5.7-fold higher than normal. In patients with an extremely high degree of hyperbilirubinemia (above 200 µmol/L), the MDA level was very high and averaged 32.3 [21.4:39.4] µmol/L, which was 11.5-fold higher than normal and significantly different from all the other subgroups of patients graded according to the level of hyperbilirubinemia.

Correlation analysis of FRP parameters with standard laboratory markers was performed. Significant direct relationships were established between the level of leukocytes with sCLII, ESR with bCLII and liver enzymes (ALT and AST) with APA. However, the strongest correlations were found between hyperbilirubinemia and the levels of bCLII, AC and MDA; the strongest was found between indicators of total and conjugated bilirubin and MDA (Table 6).

### 3.2. Predictive Values of FRP Indices Studied on the First Day of Hospitalization in Obstructive Jaundice Patients with Favorable (Discharged from Hospital) and Unfavorable (Hospital Mortality) Outcomes

An important stage in our research was the study of the prognostic significance of the FRP indicators. To do this, we divided the patients by hospital outcomes (Table 7). Seventeen cases (13.3%) of jaundice ended in mortality, including nine patients (30%) with tumorous jaundice (seven patients with pancreatic tumors and two with metastases), as well as eight patients (8.2%) with non-tumorous jaundice (five patients with jaundice caused by cholelithiasis with choledocholithiasis, two patients with chronic pathologies in the choledochal bile duct and one patient with cholangitis). The mortality rate in patients with tumorous jaundice was 3.6-fold higher (*p* < 0.05).

Mortality was typical for patients hospitalized with severe conditions. The patients with oncological obstructive jaundice who were hospitalized in a severe condition died (in all nine patients with tumorous jaundice, the SOFA score was higher than four), and six of eight (75%) patients with non-tumorous jaundice in a severe condition were hospitalized but later died.

The duration of the cholestasis symptoms in the entire sample of patients was more lethal in the case of tumorous jaundice (six of nine cases lasting more than seven days). In the case of non-tumorous jaundice, however, the patients whose symptoms of cholestasis lasted less than seven days (for two cases, the first two days; for six cases, three to six days) died. The difference was statistically significant (*p* < 0.05).

No patterns of the outcome of jaundice were found to be related to the level of total bilirubin. Fatal outcomes for tumorous jaundice with a total bilirubin level of less than 50 mmol/L at the time of admission to hospital were recorded in three cases; in six cases, the levels of bilirubin were higher than 200 mmol/L. Lethal outcomes for non-tumorous jaundice were recorded in patients whose bilirubin levels were on average lower during hospitalization (50–100 mmol/L in two cases, 100–200 mmol/L in four cases and only two cases with high hyperbilirubinemia above 200 mmol/L). Thus, the level of bilirubinemia itself is a rather weak prognostic sign.

After conducting a comparative analysis of FRP indicators in patients with lethal outcomes (*n* = 17) and favorable outcomes (111 discharged patients), the predictive value of the MDA indicator (*p* < 0.05) was established and confirmed by correlation analysis of the outcome (r = 0.223; *p* < 0.01). On average, the patients who died in the hospital demonstrated a 24% higher level of MDA during hospitalization than those who were discharged (*p* < 0.05). Quantitative MDA values were obtained and may now serve as an objective criterion for assessing the risk of an unfavorable outcome. In discharged patients with jaundice, the MDA level varied from 2.3 to 37.2 mmol/L, while in the deceased patients it ranged from 3.9 to 52.5 μmol/L. Consequently, MDA levels less than 3.9 μmol/L correspond to a favorable prognosis and a low risk of death during obstructive jaundice; MDA levels higher than 37.2 μmol/l, on the contrary, signal a very high risk of an unfavorable outcome. The same MDA levels allow early prediction of postoperative mortality.

Thus, analysis of FRP parameters on the first day of hospitalization of patients with jaundice revealed the significant role played by oxidative stress in the pathogenesis of obstructive jaundice, both non-tumorous and tumorous. The FRP parameters, especially MDA, are early markers of jaundice severity and can be used as extra diagnostic tests to assess the severity of jaundice and make a prognosis on the outcome of the disease.

### 3.3. Analysis of the Dynamics of Oxidative Stress Indicators and Perfusion Syndrome Identification after Surgically Performed Decompression

The FRP dynamics analysis (during hospitalization, on days 3, 8 and 14 after a primary decompression) made it possible to establish the presence of a long-term imbalance of oxygen and lipid components, which persisted until patients were discharged from the hospital.

The maximum FRP imbalance was recorded on the first day of hospitalization (for the lipid part of the free radical spectrum, according to the MDA indicator) and on the third day after decompression (for the oxygen part of the free radical processes, according to the AC indicator).

**Figure 6:**
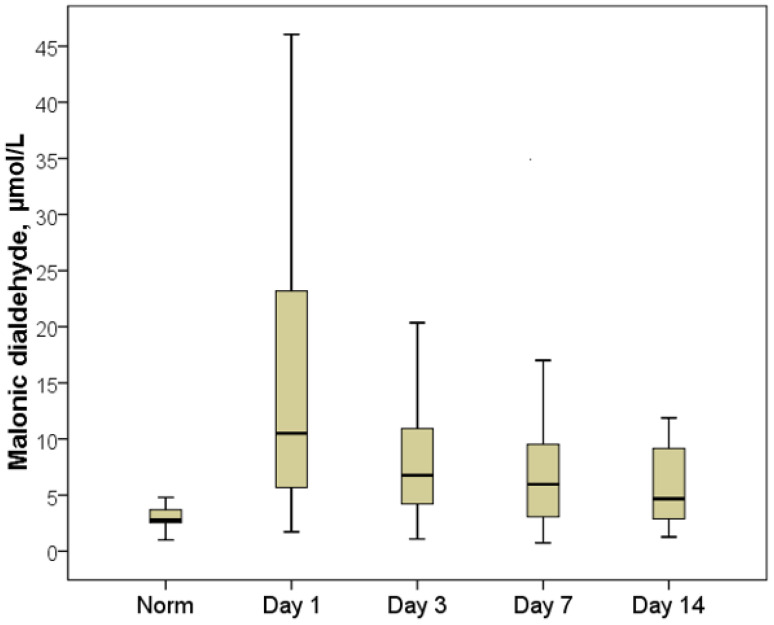
Dynamics of the malondialdehyde indicator.

**Figure 7:**
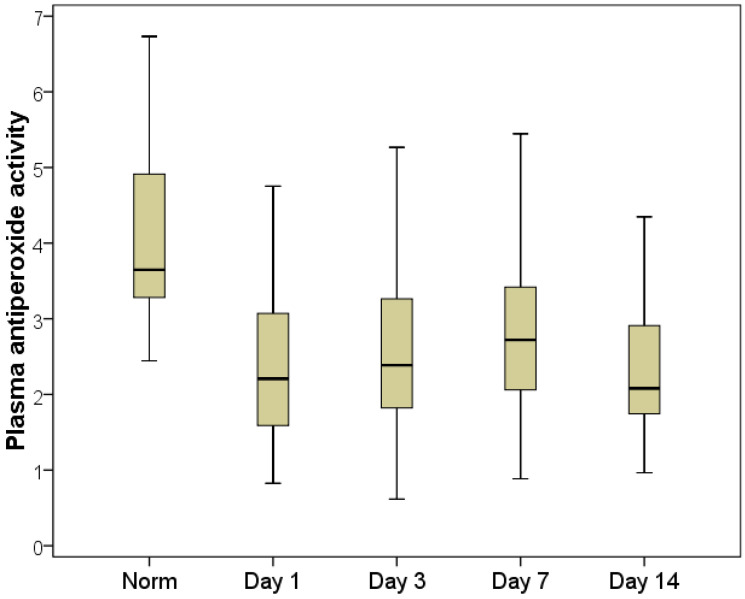
Dynamics of the APA indicator.

The plasma MDA index decreased 1.7-fold by the third day compared with the first day of hospitalization, averaging 6.8 µmol/L with an interquartile range of [4.2:10.9] µmol/L (Figure 6). Despite the fact that this indicator remained significantly higher than normal (2.5-fold on average; *p* < 0.01), in a quarter of patients, the MDA returned to normal on day three and corresponded to the normal reference values, providing evidence for the effectiveness of decompression in terms of leveling the activity of cell death and endotoxicosis. This was confirmed by an increase in the APA indices, which were on average 10% higher on the third day than on the first day. On the seventh day, the plasma APA increased by an average of 22%, and in more than half of the patients, the APA level corresponded to normal values by the seventh day (Figure 7).

It has been observed that activation of the immune system requires a significant amount of time, which means that, rather than relying solely on surgery to address the problem, conservative therapy should be chosen carefully and patients frequently and closely observed after decompression.

**Figure 8:**
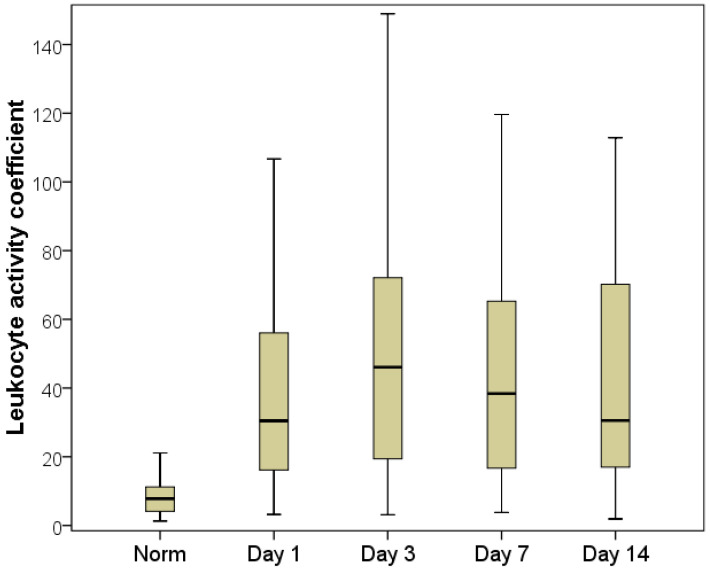
Dynamics of the AC indicator.

We also determined the intensified production of active oxygen forms by the third day after the resolution of cholestasis. This was demonstrated by the AC indicator, which was on average 1.50-fold (46.1 [19.4:72.1]) higher on the third day than on the first day (*p* < 0.01) (Figure 8). Such a significant increase in this indicator is associated with a decrease in the bCLII indicator (19% lower for day three than on the day of hospitalization (Me = 32.2 mV/s × 10^6^ L with an interquartile range of [20.2:77.7]), occurring simultaneously with an insignificant increase in the sCLII indicator (Table 8). However, the influence of the performed, albeit minimal, surgical intervention cannot be completely ignored.

The increase in the reactive oxygen species generated by leukocytes on day three can be explained by the reperfusion syndrome that accompanies a sharp drop in pressure in the biliary tree, along with operational stress and anesthesia. In patients who underwent decompression in a minimally invasive manner, there was a slow decrease in pressure in the biliary tree, so no exacerbation of the imbalance of the FRP parameters was found.

This statement is illustrated by the highest peaks in indicators for patients with prolonged jaundice after cholecystectomy with external drainage of the choledochal bile duct, when a rapid release of pressure in the gallbladder provokes a drastic discharge of toxins from the liver in response to restored hepatic blood flow and an increase in macrophage activity (AC, sCLII).

By the time of discharge from the hospital, the FRP indices had improved (on the 7th and 14th days); however, even on the last day of the study, all FRP indices were significantly divergent from normal (on the 14th day, bCLII was on average 1.77-fold lower, sCLII was 2.72-fold higher, AC was 3.87-fold higher, APA was 1.71-fold lower and MDA was 1.67-fold higher (*p* < 0.05)). This indicates long-term imbalances in the FRP and the long-term oxidative stress during obstructive jaundice of various etiologies. Therefore, it can be recommended that antioxidant therapy be implemented to improve the results of treatment of patients with benign formations, tumorous jaundice and after decompression surgery.

## 4. Discussion

The analysis of the samples obtained proved that FRP indices differ significantly between healthy individuals and patients with obstructive jaundice. Therefore, it is not surprising that, based on the earlier studies, other researchers are trying to find drugs to normalize free radical processes in patients with jaundice [31,32,33,34].

We also noted a direct correlation between FRP indicators and the duration and severity of the patient’s jaundice. Moreover, in cases of severe jaundice, we observed decreased plasma anti-peroxide activity, which has become recognized as one of the signs of a poor prognosis or slow recovery. It could be assumed that the effects of an increase in oxidative stress also depend on the type (class) of the tumor. However, a relatively small amount of material (30 patients with tumor jaundice), when broken down into smaller groups, does not allow us to come to a reliable conclusion for this hypothesis.

Therefore, the main criteria for a strong correlation between the indicators of FRP and the severity of jaundice were the values for sCLII and MDA. The same phenomenon was observed during the comparison of bilirubin levels with the severity of the disease; in fact, the abovementioned FRP indicators often ‘outpaced’ the usual bilirubin indicators, which have come to be seen as unreliable prognostic markers. The decrease in bilirubin levels after decompression surgeries often outpaced the dynamics of FRP, demonstrating a slow recovery from the pathophysiological processes that began in the body.

Comparative analysis of FRP in patients with jaundice of a benign or malignant genesis revealed that the greatest FRP imbalance occurred in cases of tumorous jaundice. A significant difference in the MDA level was also recorded. However, the duration and severity of jaundice as a cause of these figures cannot be excluded. This was confirmed by the FRP-level analysis for various durations of jaundice. During the analysis, no significant differences were observed in the rate of liver failure, marked by the death of hepatocytes, as determined based on the MDA levels. An increase in MDA, a marker of intoxication and cell death, was registered from the first day of clinically significant jaundice, which affirmed the results of experimental work carried out by other researchers [35]. This confirms the need for biliary relief for jaundice patients as soon as possible and is consistent with the standard of treatment for jaundice accepted by surgeons.

The results of our study also showed that even an indicator of FRP, such as plasma MDA, could itself be a reliable early marker of the disease severity and outcome. Without a doubt, lipid peroxidation has a direct effect on the formation of complexes consisting of protein molecules and oxidized lipids, as well as the polymerization of phospholipids and polypeptides, thereby changing the structure, and hence the function, of the cell membrane itself. These protein-lipid complexes often form strong chemical bonds between the free amino groups of amino acid radicals and the aldehyde (or carboxyl) groups of oxidized lipids. The most significant role in the formation of these complexes is played by MDA, which as one of the end-products of the lipid peroxidation process, forms Schiff bases and reacts with lysine ε-amino groups or residues of N-terminal amino acids in polypeptide molecules [36,37,38]. Therefore, measuring MDA can be recommended as an additional diagnostic test for assessing the severity of and prognosis for jaundice. In addition, the indicators characterizing free radical processes should be used to determine the area of treatment, the amount of surgical treatment and to make decisions about immediate hospitalization in the intensive care unit.

Thus, the data obtained in patients with obstructive jaundice have again confirmed the general pathophysiological mechanism responsible for the development of organ lesions in this pathology. These data lend support to the urgent need for antioxidant therapy starting from the first day of hospitalization as an important component of pathogenetic therapy in the treatment of patients with jaundice of varied genesis and severity.

Development of a specific drug for the treatment of jaundice, judging by the published works, is far from complete, nor simple, and requires further research.

The authors hope that further research, as material is collected, will provide an opportunity to find additional signs, markers and correlations, as well as pattern consistency in free radical processes in different types of tumors, including in Gi cancer. This will improve the outcome of treatment of patients with tumor and non-tumor obstructive jaundice.

## 5. Conclusions

Oxidative stress plays a significant role in the pathogenesis of tumorous and non-tumorous obstructive jaundice and persists in jaundice of different etiologies up to and including when patients are discharged from the hospital (up to 14 days).

Surgical treatment of jaundice, on the one hand, leads to a significant decrease in markers of lipid peroxidation (MDA), demonstrating clear benefits by preserving cells and inhibiting intoxication and hepatocellular failure processes; on the other hand, it is accompanied by intensified generation of reactive oxygen species by blood leukocytes within three days, which may be a manifestation of the reperfusion syndrome that occurs after decompression of the bile tree.

Therefore, antioxidant therapy can be useful for improving the results of treatment of patients with benign and malignant jaundice.

Parameters of FRP, especially MDA, are early markers of the disease severity and can be used as additional diagnostic tests to assess the severity and prognosis of jaundice.

## Figures and Tables

**Table 1:** Distribution of patients by sex, age, duration and severity of tumorous and non-tumorous jaundice.

Characteristic	Benign Jaundice (*n* = 98)	Tumor Jaundice (*n* = 30)	*p*	Total (*n* = 128)
Sex				
Male	44 (44.9%)	10 (33.3%)	0.182	54 (42.2%)
Female	54 (55.1%)	20 (66.7%)		74 (57.8%)
Age				
<50 years	12 (12.2%)	1 (3.3%)	0.070	13 (10.2%)
50–64 years	24 (24.5%)	4 (13.3%)		28 (21.9%)
65–79 years	36 (36.7%)	15 (50.0%)		51 (39.8%)
≥80 years	26 (26.5%)	10 (33.3%)		36 (28.1%)
Distribution of patients by the severity of the condition (via the SOFA scale) at the time of admission				
Non-severe; <4 points (*n*; %)	69 (70.4%)	8 (26.7%)	0.0001	77 (60.2%)
Severe ≥4 points (*n*; %)	29 (29.6%)	22 (73.3%)		51 (39.8%)
Duration of jaundice (according to patients’ accounts) at the time of admission (days)				
0–2 days	41 (41.8%)	3 (10.0%)	0.0001	44 (34.4%)
3–6 days	42 (42.9%)	10 (33.3%)		52 (40.6%)
≥7 days	15 (15.3%)	17 (56.7%)		32 (25.0%)
Distribution of patients according to the severity of jaundice at the time of admission (according to the total level of bilirubin (μmol/L))				
<100	43 (43.9%)	7 (23.3%)	0.0001	50 (39.1%)
100–150	32 (32.7%)	4 (13.3%)		36 (28.1%)
>150	23 (23.5%)	19 (63.3%)		42 (32.8%)
Treatment types				
Conservative treatment	22 (22.4%)	6 (20.0%)	0.0001	28 (21.9%)
Endoscopic papilo-sphincterotomy (EPST)	57 (58.2%)	0		57 (44.5%)
Microcholecystostomy (gall bladder puncture)	6 (6.1%)	9 (30.0%)		15 (11.7%)
Cholecystectomy (drainage of the bile duct)	9 (9.2%)	8 (26.7%)		17 (13.3%)
Nasobiliary drainage, stenting	4 (4.1%)	7 (23.3%)		11 (8.6%)
Treatment				
Conservative therapy	22 (22.4%)	6 (20.0%)	0.598	28 (21.9%)
Surgical intervention	76 (77.6%)	24 (80.0%)		100 (78.1%)

**Table 2:** Indicators of free radical processes in patients with obstructive jaundice during hospitalization.

Indicators	Norm (*n* = 33)	Obstructive Jaundice (*n* = 128)	*p* (Mann-Whitney)
bCLII (mV/s × 10^6^ L)	62.5 [44.9:79.3]	40.2 [24.5:76.9]	0.040
sCLII (mV/s × 10^6^ L)	465.5 [317.6:516.4]	1507 [804:2745]	<0.001
AC (sCLII/bCLII)	7.9 [4.3:11.4]	30.7 [16.1:56.4]	<0.001
APA (RU)	3.6 [3.3:4.9]	2.2 [1.6:3.1]	<0.001
MDA (μmol/L)	2.8 [2.5:3.6]	10.5 [5.7:23.2]	<0.001

Note: The table represents the medians and interquartile ranges of the indicators.

**Table 3:** Indicators of free radical processes in patients with jaundice of a tumorous or non-tumorous etiology.

Obstructive Jaundice	bCLII (mV/s × 10^6^ L)	sCLII (mV/s×10^6^ L)	AC (sCLII/bCLII)	APA (RU)	MDA (μmol/L)
Non-tumorous (*n* = 98)	45.2 [24.7:84.4]	1592 ** [945:2674]	30.6 ** [16.5:54.9]	2.1 ** [1.6:2.9]	8.9 ** [5.6:17.2]
Tumorous (*n* = 30)	28.5 ** [18.8:57.6]	1398 ** [689:3269]	31.5 ** [14.6:57.3]	2.3 * [1.6:3.3]	24.1 ** [9.5:32.3]
*p*	0.162	0.559	0.842	0.645	0.003

Note: Medians and interquartile ranges of the indicators are shown. * Deviation from normal at *p* < 0.05; ** *p* < 0.01.

**Table 4:** Distribution of patients by severity of disease and level of hyperbilirubinemia (*p* < 0.001).

Severity via SOFA Scale	Total Bilirubin Level (μmol/L)				Total
	Under 50	50–100	100–200	>200	
Non-severe (<4)	22 (84.6%)	29 (76.3%)	25 (64.6%)	1 (3.7%)	77 (60.2%)
Severe (≥4)	4 (15.4%)	9 (23.7%)	12 (32.4%)	26 (96.3%)	51 (39.8%)

**Table 5:** Indicators of free radical processes in patients with obstructive jaundice of varying degrees of severity.

	bCLII (mV/s × 10^6^ L)	sCLII (mV/s × 10^6^ L)	AC (sCLII/bCLII)	APA (RU)	MDA (μmol/L)
Not severe obstructive jaundice	35.8 * [23.9:68.5]	1488 ** [757:2451]	30.6 ** [16.9:56.4]	2.3 * [1.7:3.1]	8.8 ** [5.4:15.9]
Severe obstructive jaundice	57.6 [27.9:83.9]	1742 ** [839:3303]	30.9 ** [15.4:55.9]	1.9 ** [1.5:2.9]	19.1 ** [8.1:32.9]
*p* (Mann-Whitney)	0.071	0.158	0.721	0.258	0.001

Note: Medians and interquartile ranges of indicators are shown. * Statistically significant difference in comparison with normal at *p* < 0.05; ** at *p* < 0.01.

**Table 6:** Correlation analysis of free radical processes’ indicators with indicators of bilirubin, AST, ALT, the number of leukocytes and ESR examined in the blood of patients with jaundice on the first day of hospitalization (the correlation matrix contains only reliable relationships with a strength higher than 0.2).

Indicators	Total Bilirubin	Conjugated Bilirubin	Unconjugated Bilirubin	ALT	AST	Alkaline Phosphatase	Leukocytes	ESR
bCLII								0.253 *
sCLII	0.270 **	0.317 **					0.290 **	
AC	0.355 **	0.374 **	0.250 *					
APA				0.401 **	0.452 **			
MDA	0.740 **	0.705 **	0.594 **			0.225 **		

Note: * *p* < 0.05; ** *p* < 0.01.

**Table 7:** Outcomes of tumorous and non-tumorous jaundice.

Outcomes	Benign Diseases (*n* = 98)	Tumors (*n* = 30)	*p*	Total (*n* = 128)
Healed	36 (36.7%)	0	0.0001	36 (28.1%)
Improved condition	51 (52.0%)	20 (66.7%)		71 (55.5%)
Discharged, severe condition	3 (3.1%)	1 (3.3%)		4 (3.1%)
Deceased	8 (8.2%)	9 (30.0%)		17 (13.3%)

**Table 8:** Dynamics of FRP indicators.

Day	MDA (Norm: 1.5−4.1; Me = 2.75)	APA (Norm: 2.7−6.0; Me = 3.6)	bCLII (Norm: 31−105; Me = 62.5)	sCLII (Norm: 140−700; Me = 465)	AC (Norm: 2.0−15.4; Me = 7.9)
1	10.5 * [5.7:23.2]	2.2 * [1.6:3.1]	40.2 * [24.5:76.9]	1507 * [804:2745]	30.7 * [16.1:56.4]
3	6.8 * [4.2:10.9]	2.4 * [1.8:3.3]	32.2 * [20.2:77.7]	1536 * [872:2404]	46.1 * [19.4:72.1]
7	6.0 * [3.1:9.5]	2.7 * [2.1:3.4]	34.6 * [16.5:73.7]	1368 * [669:1862]	38.4 * [16.7:65.3]
14	4.6 * [2.9:9.2]	2.1 * [1.7:2.9]	35.1 * [17.3:74.2]	1264 * [628:2056]	30.6 * [17.0:70.2]

Note: Medians and interquartile ranges of indicators are shown. * Statistically significant difference in comparison with normal at *p* < 0.05.

## Data Availability

The database and the indicators analyzed in the work are held by the main authors, Ekaterina Silina (silinaekaterina@mail.ru) and Victor Stupin (stvictor@bk.ru).
